# Supplementary material for: Multi-component generalized mode-coupling theory: predicting dynamics from structure in glassy mixtures
Source: Eur Phys J E Soft Matter. 2021 Jul 6;44(7):91. doi: 10.1140/epje/s10189-021-00095-w (PMC8260512; doi:10.1140/epje/s10189-021-00095-w)
Supplement: Supplementary file 1 — Supplementary material 1 (pdf 783 KB) [file 10189_2021_95_MOESM1_ESM.pdf]

Multi-component generalized mode-coupling theory: Predicting dynamics from structure in glassy mixtures

Simone Ciarella,[1,2,*] Chengjie Luo,[1,*] Vincent E. Debets,[1,*] and Liesbeth M. C. Janssen[1]

[1]*Soft Matter and Biological Physics, Department of Applied Physics, Eindhoven University of Technology, P.O. Box 513, 5600 MB Eindhoven, The Netherlands*

[2]*Laboratoire de Physique de l'Ecole Normale Supérieure, ENS, Université PSL, CNRS, Sorbonne Université, Université de Paris, F-75005 Paris, France*

---

[*] These authors contributed equally to this work

I. SUPPLEMENTARY INFORMATION

A. Derivation of multi-component GMCT

In this section we present the full derivation of multi-component GMCT. We first introduce the Mori-Zwanzig formalism, which can be used to obtain exact dynamical equations for the correlation functions of arbitrary vectors of classical variables. Choosing the vector to consist of 1-point (species dependent) density and current modes, we rederive standard multi-component MCT and highlight several technical details which will also prove to be useful for the derivation of GMCT. Finally, we generalize the vector to include multi-point density and current modes and retrieve the microscopic time-dependent multi-component GMCT equations.

1. Mori-Zwanzig formalism

Any dynamical vector $\boldsymbol{A}(t)$, whose elements $A_i(t) = [\boldsymbol{A}(t)]_i$ are functions of classical phase space variables, changes over time according to $\frac{d\boldsymbol{A}(t)}{dt} = \{\boldsymbol{A}(t), \mathcal{H}\} = i\mathcal{L}\boldsymbol{A}(t)$ [1]. Here, $\{\cdots\}$ denotes the Poisson bracket, $\mathcal{H}$ is the Hamiltonian of the system, and $\mathcal{L}$ depicts the classical Liouville operator. In analogy to vector algebra, one can define a projection operator P to project onto the space spanned by $\boldsymbol{A} = \boldsymbol{A}(0)$:

$$P = \sum_{ij} |A_i) T_{ij} (A_j|. \tag{1}$$

Using the idempotent property $PP = P$, the matrix $\boldsymbol{T}$ can be determined via

$$\sum_j T_{ij} (A_j | A_l) = \delta_{il}, \tag{2}$$

where $(X|Y) = \langle X^* Y \rangle$ defines a scalar product of variables X and Y, $\langle \cdots \rangle$ is the ensemble average, and δ_{il} is the Kronecker delta function. If we denote $\boldsymbol{G} = (\boldsymbol{A}|\boldsymbol{A})$ or $G_{ij} = [\boldsymbol{G}]_{ij} = (A_i|A_j)$, then

$$\boldsymbol{T} = \boldsymbol{G}^{-1}. \tag{3}$$

Usually, though not necessarily, the elements in the vector $\boldsymbol{A}$ are chosen to be slow or quasi-conserved variables of the system. As a result, a projection only retains the slow part that is parallel to $\boldsymbol{A}$, and it removes the orthogonal or fast part, which can be obtained by using the complementary operator $1 - P$. Inserting several of those projections, the dynamical equation of $\boldsymbol{A}(t)$ can be written as

$$\frac{d\boldsymbol{A}(t)}{dt} = e^{i\mathcal{L}t}(P + 1 - P) i\mathcal{L}\boldsymbol{A} = e^{i\mathcal{L}t} P i\mathcal{L}\boldsymbol{A} + e^{i\mathcal{L}t}(1 - P) i\mathcal{L}\boldsymbol{A} = e^{i\mathcal{L}t} P \dot{\boldsymbol{A}} + e^{i\mathcal{L}t}(1 - P)\dot{\boldsymbol{A}} \tag{4}$$

If we then replace $e^{i\mathcal{L}t}$ in the last term by the identity [2]

$$e^{i\mathcal{L}t} = \int_0^t d\tau e^{i\mathcal{L}(t-\tau)} iP\mathcal{L} e^{i(1-P)\mathcal{L}\tau} + e^{i(1-P)\mathcal{L}t}, \tag{5}$$

we obtain

$$|\dot{A}_k(t)) = \sum_{ij} |A_i(t)) (\boldsymbol{G}^{-1})_{ij} (A_j | \dot{A}_k) - \int d\tau \sum_{ij} |A_i(t-\tau)) (\boldsymbol{G}^{-1})_{ij} \left(f_j | f_k(\tau) \right) + |f_k(t)), \tag{6}$$

where we have introduced the so-called fluctuating force

$$|f_k(t)) \equiv e^{i(1-P)Lt} (1 - P) |\dot{A}_k), \tag{7}$$

which, at $t = 0$, evolves in time according to

$$|f_k) = |\dot{A}_k) - \sum_{ij} |A_i) (\boldsymbol{G}^{-1})_{ij} (A_j | \dot{A}_k). \tag{8}$$

Physically, the fluctuating force represents the time evolution in the subspace orthogonal to $\boldsymbol{A}$, i.e., it constitutes the fast part of the dynamics. It should therefore be orthogonal to the slow variable, i.e. $(A_i | f_k(t)) = 0$, which is easily

checked from the definition of $|f_k(t))$. Invoking this orthogonality, we may find the following dynamical equation for the correlation functions $C_{ij}(t) = (A_i|A_j(t))$,

$$\dot{C}_{ij}(t) = \sum_{lm} C_{il}(t)(\boldsymbol{G}^{-1})_{lm}(A_m|\dot{A}_j) - \int d\tau \sum_{lm} C_{il}(t-\tau)(\boldsymbol{G}^{-1})_{lm}(f_m|f_j(\tau)). \tag{9}$$

Note that in this formalism, correlation functions are also written as scalar products. We point out that the above equation is exact and that the main difficulty of calculating the correlation functions $C_{ij}(t)$ resides in finding an (approximate) expression for the memory function $K_{mj}(\tau) = (f_m|f_j(\tau))$. The procedure above, which effectively separates the dynamics of a system into a relevant (slow) and irrelevant (fast) part, is called the Mori-Zwanzig formalism and both MCT and GMCT are based on it.

2. *Multi-component MCT*

Now let us focus on a multi-component model system consisting of N particles and M species. For such a system we will choose as our slow variables

$$\boldsymbol{A} = [\{\rho^\alpha_{\boldsymbol{k}}\}_{\alpha\in\{1,2,\dots,M\}}, \{j^\alpha_{\boldsymbol{k}}\}_{\alpha\in\{1,2,\dots,M\}}], \tag{10}$$

where $\rho^\alpha_{\boldsymbol{k}} = \sum_{i=1}^{N_\alpha} e^{i\boldsymbol{k}\cdot\boldsymbol{r}_i}/\sqrt{N}$ is a density mode for species α, $j^\alpha_{\boldsymbol{k}} = -i\dot{\rho}^\alpha_{\boldsymbol{k}}$ is the corresponding current mode, and the wavevector $\boldsymbol{k}$ probes the length scale of interest. The matrix C_{ij} can then be regarded as four blocks, written schematically as $\begin{bmatrix} C_{11} = (\rho|\rho) & C_{12} = (\rho|j) \\ C_{21} = (j|\rho) & C_{22} = (j|j) \end{bmatrix}$, although we will primarily focus on the dynamics of the upper-left block with elements $(\rho^\alpha_{\boldsymbol{k}}|\rho^\beta_{\boldsymbol{k}}(t))$. Making use of the fact that $(\rho^\alpha_{\boldsymbol{k}}|\rho^\beta_{\boldsymbol{k}}) = S_{\alpha\beta}(k)$, $(\dot{\rho}^\alpha_{\boldsymbol{k}}|\dot{\rho}^\beta_{\boldsymbol{k}}) = \delta_{\alpha\beta}k^2\frac{k_BT}{m_\alpha}x_\alpha \equiv J_{\alpha\beta}(k)$, and $(\rho^\alpha_{\boldsymbol{k}}|\dot{\rho}^\beta_{\boldsymbol{k}}) = 0$, it is easy to show that

$$\boldsymbol{G} = \begin{bmatrix} \boldsymbol{S}(k) & \boldsymbol{0} \\ \boldsymbol{0} & \boldsymbol{J}(k) \end{bmatrix}, \tag{11}$$

and

$$(\boldsymbol{A}|\dot{\boldsymbol{A}}) = \begin{bmatrix} \boldsymbol{0} & i\boldsymbol{J}(k) \\ i\boldsymbol{J}(k) & \boldsymbol{0} \end{bmatrix}. \tag{12}$$

with $\boldsymbol{S}$ and $\boldsymbol{J}$ depicting $M \times M$ matrices. Moreover, the fluctuating force can be calculated to give

$$\boldsymbol{f} = \left[\{\dot{\rho}^\alpha_{\boldsymbol{k}}\}, \{\dot{j}^\alpha_{\boldsymbol{k}}\}\right] - \left[\{\rho^\alpha_{\boldsymbol{k}}\}, \{j^\alpha_{\boldsymbol{k}}\}\right] \begin{bmatrix} \boldsymbol{S}^{-1}(k) & \boldsymbol{0} \\ \boldsymbol{0} & \boldsymbol{J}^{-1}(k) \end{bmatrix} \begin{bmatrix} \boldsymbol{0} & i\boldsymbol{J}(k) \\ i\boldsymbol{J}(k) & \boldsymbol{0} \end{bmatrix} \tag{13}$$

$$= \left[\{\dot{\rho}^\alpha_{\boldsymbol{k}}\}, \{\dot{j}^\alpha_{\boldsymbol{k}}\}\right] - \left[i\{j^\alpha_{\boldsymbol{k}}\}, i\{\rho^\alpha_{\boldsymbol{k}}\}\boldsymbol{S}^{-1}(k)\boldsymbol{J}(k)\right] \tag{14}$$

$$= \left[\boldsymbol{0}, \{\dot{j}^\alpha_{\boldsymbol{k}}\} - i\{\rho^\alpha_{\boldsymbol{k}}\}\boldsymbol{S}^{-1}(k)\boldsymbol{J}(k)\right], \tag{15}$$

which implies that each element of $\boldsymbol{f}$ yields $f^\alpha_k = \dot{j}^\alpha_{\boldsymbol{k}} - i\sum_{\beta\gamma}\rho^\beta_{\boldsymbol{k}}\left(S^{-1}(k)\right)_{\beta\gamma}J_{\gamma\alpha}(k)$. Using these ingredients allows us to write down a dynamical equation for $F_{\alpha\beta}(\boldsymbol{k},t) = (\rho^\alpha_{\boldsymbol{k}}|\rho^\beta_{\boldsymbol{k}}(t))$ in the following manner:

$$\ddot{F}_{\alpha\beta}(\boldsymbol{k},t) + \sum_{\gamma\theta} F_{\alpha\gamma}(\boldsymbol{k},t)\left(\boldsymbol{S}^{-1}(k)\right)_{\gamma\theta} J_{\theta\beta}(k) + \int d\tau \dot{F}_{\alpha\gamma}(\boldsymbol{k},t-\tau)\left(\boldsymbol{J}^{-1}(k)\right)_{\gamma\theta} K_{\theta\beta}(\boldsymbol{k},\tau) = 0. \tag{16}$$

As stated previously, the memory function $K_{\theta\beta}(\boldsymbol{k},\tau) = (f^\theta_{\boldsymbol{k}}|f^\beta_{\boldsymbol{k}}(\tau))$ forms the main problem for any analytical progress. We therefore seek to approximate it by inserting the projection operator

$$P_2 = \sum_{\alpha'\beta'\boldsymbol{q}'\boldsymbol{p}'}^{\alpha\beta\boldsymbol{q}\boldsymbol{p}} |\rho^\alpha_{\boldsymbol{q}}\rho^\beta_{\boldsymbol{p}}) T^{\alpha\beta\boldsymbol{q}\boldsymbol{p}}_{\alpha'\beta'\boldsymbol{q}'\boldsymbol{p}'} (\rho^{\alpha'}_{\boldsymbol{q}'}\rho^{\beta'}_{\boldsymbol{p}'}| \tag{17}$$

in front of the fluctuating force and replacing the projected time evolution by a full one, i.e. $(f^\theta_{\boldsymbol{k}}|f^\beta_{\boldsymbol{k}}(\tau)) \approx (f^\theta_{\boldsymbol{k}}P_2|e^{iLt}|P_2 f^\beta_{\boldsymbol{k}})$. The first part of this approximation, i.e. applying the projector P_2, is rooted in the assumption that the dominant contributions to $f^\theta_{\boldsymbol{k}}$ arise from slow pair-density modes $\rho^\alpha_{\boldsymbol{q}}\rho^\beta_{\boldsymbol{p}}$. The replacement of the projected time evolution operator $e^{i(1-P)Lt}$ in the memory function by e^{iLt} is mainly to keep calculations tractable.

Note that we have introduced the most general definition of P_2 in which all wavevectors and species of the involved density modes can be different. However, such a strict definition will prove not to be necessary, since the wavevectors are constrained due to translational invariance of the system and a coupling to the wavevector $\boldsymbol{k}$ in $f^\theta_{\boldsymbol{k}}$ [2]. How this simplifies P_2 will become more apparent in the following parts of the derivation of MCT. We proceed by first specifying the normalization of P_2. Using the property $P_2 P_2 = P_2$ gives

$$\sum_{\alpha'\beta'\boldsymbol{q}'\boldsymbol{p}'} T^{\alpha\beta\boldsymbol{q}\boldsymbol{p}}_{\alpha'\beta'\boldsymbol{q}'\boldsymbol{p}'} (\rho^{\alpha'}_{\boldsymbol{q}'} \rho^{\beta'}_{\boldsymbol{p}'} | \rho^{\gamma}_{\boldsymbol{m}} \rho^{\theta}_{\boldsymbol{n}}) = \delta_{\alpha\gamma}\delta_{\beta\theta}\delta_{\boldsymbol{q}\boldsymbol{m}}\delta_{\boldsymbol{p}\boldsymbol{n}}, \tag{18}$$

where, under the assumption of Gaussian factorization, we have

$$(\rho^{\alpha'}_{\boldsymbol{q}'} \rho^{\beta'}_{\boldsymbol{p}'} | \rho^{\gamma}_{\boldsymbol{m}} \rho^{\theta}_{\boldsymbol{n}}) \approx S^{\alpha'\gamma}_m \delta_{q'm} S^{\beta'\theta}_n \delta_{p'n} + S^{\alpha'\theta}_n \delta_{q'n} S^{\beta'\gamma}_m \delta_{p'm}, \tag{19}$$

such that

$$\sum_{\alpha'\beta'} T^{\alpha\beta\boldsymbol{q}\boldsymbol{p}}_{\alpha'\beta'\boldsymbol{m}\boldsymbol{n}} S^{\alpha'\gamma}_m S^{\beta'\theta}_n + \sum_{\alpha'\beta'} T^{\alpha\beta\boldsymbol{q}\boldsymbol{p}}_{\alpha'\beta'\boldsymbol{n}\boldsymbol{m}} S^{\alpha'\theta}_n S^{\beta'\gamma}_m = \delta_{\alpha\gamma}\delta_{\beta\theta}\delta_{\boldsymbol{q}\boldsymbol{m}}\delta_{\boldsymbol{p}\boldsymbol{n}}. \tag{20}$$

Then, making use of the fact that α' and β' in the second term on the left-hand side are interchangeable and that $T^{\alpha\beta\boldsymbol{q}\boldsymbol{p}}_{\alpha'\beta'\boldsymbol{m}\boldsymbol{n}} = T^{\alpha\beta\boldsymbol{q}\boldsymbol{p}}_{\beta'\alpha'\boldsymbol{n}\boldsymbol{m}}$ by symmetry, we are allowed to rewrite

$$2\sum_{\alpha'\beta'} T^{\alpha\beta\boldsymbol{q}\boldsymbol{p}}_{\alpha'\beta'\boldsymbol{m}\boldsymbol{n}} S^{\alpha'\gamma}_m S^{\beta'\theta}_n = \delta_{\alpha\gamma}\delta_{\beta\theta}\delta_{\boldsymbol{q}\boldsymbol{m}}\delta_{\boldsymbol{p}\boldsymbol{n}}, \tag{21}$$

which defines our normalization tensor $T^{\alpha\beta\boldsymbol{q}\boldsymbol{p}}_{\alpha'\beta'\boldsymbol{m}\boldsymbol{n}}$. Having fully specified our operator, we now seek to calculate the projected force $P_2|f^\mu_{\boldsymbol{k}}) = P_2\left[|\dot{j}^\mu_{\boldsymbol{k}}) - i\sum_{\theta\phi}|\rho^\theta_{\boldsymbol{k}})\left(S^{-1}(k)\right)_{\theta\phi} J_{\phi\mu}(k)\right]$. In particular, we have

$$\begin{aligned}
&(\rho^{\alpha'}_{\boldsymbol{q}'} \rho^{\beta'}_{\boldsymbol{p}'} | \dot{j}^\mu_{\boldsymbol{k}}) = -(\dot{\rho}^{\alpha'}_{\boldsymbol{q}'} \rho^{\beta'}_{\boldsymbol{p}'} | j^\mu_{\boldsymbol{k}}) - (\rho^{\alpha'}_{\boldsymbol{q}'} \dot{\rho}^{\beta'}_{\boldsymbol{p}'} | j^\mu_{\boldsymbol{k}}) \\
&= i\frac{1}{\sqrt{N}}\frac{k_B T}{m_\mu}\delta_{\boldsymbol{q}',\boldsymbol{k}-\boldsymbol{p}'}\left[\boldsymbol{q}'\cdot\boldsymbol{k}S^{\alpha'\beta'}_{p'}\delta_{\mu\alpha'} + \boldsymbol{p}'\cdot\boldsymbol{k}S^{\alpha'\beta'}_{q'}\delta_{\mu\beta'}\right],
\end{aligned} \tag{22}$$

and, using the equation from the generalized Ornstein-Zernike relation that factorizes static triplet correlations into pair correlations with the direct triplet correlation functions c_3 as corrections [3, 4],

$$S^{\alpha'\beta'\theta}_3(\boldsymbol{q},\boldsymbol{p}',\boldsymbol{k}') = (\rho^{\alpha'}_{\boldsymbol{q}} \rho^{\beta'}_{\boldsymbol{p}'} | \rho^{\theta}_{\boldsymbol{k}}) = \delta_{\boldsymbol{q}+\boldsymbol{p}',\boldsymbol{k}}\frac{1}{\sqrt{N}}\sum_{\epsilon\sigma\eta} S^{\alpha'\epsilon}_q S^{\beta'\sigma}_{p'} S^{\theta\eta}_k\left[\delta_{\epsilon\sigma}\delta_{\sigma\eta}\delta_{\epsilon\eta}/x^2_\epsilon + \rho^2 c^{\epsilon\sigma\eta}_3(\boldsymbol{q},\boldsymbol{p}')\right], \tag{23}$$

we may also obtain

$$\begin{aligned}
&-i(\rho^{\alpha'}_{\boldsymbol{q}'} \rho^{\beta'}_{\boldsymbol{p}'} | \sum_{\theta\phi}|\rho^\theta_{\boldsymbol{k}})\left(S^{-1}(k)\right)_{\theta\phi} J_{\phi\mu}(k)) \\
&= -i\delta_{\boldsymbol{q}'+\boldsymbol{p}',\boldsymbol{k}}\frac{1}{\sqrt{N}}\frac{k_B T}{m_\mu}\left[S^{\alpha'\mu}_{q'} S^{\beta'\mu}_{p'} k^2/x_\mu + \sum_{\epsilon\sigma} S^{\alpha'\epsilon}_{q'} S^{\beta'\sigma}_{p'}\rho^2 c^{\epsilon\sigma\mu}_3(\boldsymbol{q}',\boldsymbol{p}')k^2 x_\mu\right].
\end{aligned} \tag{24}$$

Combining Eqs. (22) and (24), we arrive at

$$\begin{aligned}
&(\rho^{\alpha'}_{\boldsymbol{q}'} \rho^{\beta'}_{\boldsymbol{p}'} | f^\mu_{\boldsymbol{k}}) \\
&= -i\delta_{\boldsymbol{q}'+\boldsymbol{p}',\boldsymbol{k}}\frac{\rho}{\sqrt{N}}\frac{k_B T}{m_\mu}\left[\boldsymbol{q}'\cdot\boldsymbol{k}S^{\beta'\mu}_{p'}\sum_\theta S^{\alpha'\theta}_{q'} c^{\theta\mu}_{q'} + \boldsymbol{p}'\cdot\boldsymbol{k}S^{\alpha'\mu}_{q'}\sum_\theta S^{\beta'\theta}_{p'} c^{\theta\mu}_{p'} + \sum_{\epsilon\sigma} S^{\alpha'\epsilon}_{q'} S^{\beta'\sigma}_{p'}\rho c^{\epsilon\sigma\mu}_3(\boldsymbol{q}',\boldsymbol{p}')k^2 x_\mu\right],
\end{aligned} \tag{25}$$

where $c^{\alpha\beta}_q = (\delta_{\alpha\beta}/x_\alpha - \left(\boldsymbol{S}^{-1}(q)\right)_{\alpha\beta})/\rho$ is the direct correlation function. An inspection of Eq. (25) now clearly shows the wavevector constraint on the pair density modes produced by projecting on $|f^\mu_{\boldsymbol{k}})$, i.e. only terms $(\rho^{\alpha'}_{\boldsymbol{q}'}\rho^{\beta'}_{\boldsymbol{k}-\boldsymbol{q}'}|$ for a given $\boldsymbol{k}$ contribute to the projected fluctuating force. In other words, we can equally define the projection operator as $P_2 = \sum^{\alpha\beta\boldsymbol{q}}_{\alpha'\beta'\boldsymbol{q}'}|\rho^\alpha_{\boldsymbol{q}}\rho^\beta_{\boldsymbol{k}-\boldsymbol{q}})T^{\alpha\beta\boldsymbol{q}}_{\alpha'\beta'\boldsymbol{q}'}(\rho^{\alpha'}_{\boldsymbol{q}'}\rho^{\beta'}_{\boldsymbol{k}-\boldsymbol{q}'}|$ instead of the most general one [Eq. (17)]. Utilizing our results we finally

obtain for the full projection

$$P_2|f_{\boldsymbol{k}}^{\mu}) = \sum_{\alpha'\beta'\boldsymbol{q}'\boldsymbol{p}'}^{\alpha\beta\boldsymbol{q}\boldsymbol{p}} |\rho_{\boldsymbol{q}}^{\alpha}\rho_{\boldsymbol{p}}^{\beta})T_{\alpha'\beta'\boldsymbol{q}'\boldsymbol{p}'}^{\alpha\beta\boldsymbol{q}\boldsymbol{p}}(\rho_{\boldsymbol{q}'}^{\alpha'}\rho_{\boldsymbol{p}'}^{\beta'}|f_{\boldsymbol{k}}^{\mu}) \tag{26}$$

$$= -i\sum_{\alpha'\beta'\boldsymbol{q}'\boldsymbol{p}'}^{\alpha\beta\boldsymbol{q}\boldsymbol{p}} |\rho_{\boldsymbol{q}}^{\alpha}\rho_{\boldsymbol{p}}^{\beta})T_{\alpha'\beta'\boldsymbol{q}'\boldsymbol{p}'}^{\alpha\beta\boldsymbol{q}\boldsymbol{p}}\delta_{\boldsymbol{q}'+\boldsymbol{p}',\boldsymbol{k}}\frac{\rho}{\sqrt{N}}\frac{k_BT}{m_\mu}$$

$$\times\left[\boldsymbol{q}'\cdot\boldsymbol{k}S_{p'}^{\beta'\mu}\sum_{\theta}S_{q'}^{\alpha'\theta}c_{q'}^{\theta\mu} + \boldsymbol{p}'\cdot\boldsymbol{k}S_{q'}^{\alpha'\mu}\sum_{\theta}S_{p'}^{\beta'\theta}c_{p'}^{\theta\mu} + \sum_{\epsilon\sigma}S_{q'}^{\alpha'\epsilon}S_{p'}^{\beta'\sigma}\rho c_3^{\epsilon\sigma\mu}(\boldsymbol{q}',\boldsymbol{p}')k^2x_\mu\right]$$

$$= -\frac{i}{2}\sum_{\boldsymbol{q}'\boldsymbol{p}'}^{\alpha\beta\boldsymbol{q}\boldsymbol{p}} |\rho_{\boldsymbol{q}}^{\alpha}\rho_{\boldsymbol{p}}^{\beta})\delta_{\boldsymbol{q}'+\boldsymbol{p}',\boldsymbol{k}}\frac{\rho}{\sqrt{N}}\frac{k_BT}{m_\mu}$$

$$\times\left[\boldsymbol{q}'\cdot\boldsymbol{k}\sum_{\theta}c_{q'}^{\theta\mu}\delta_{\alpha\theta}\delta_{\beta\mu}\delta_{\boldsymbol{q}\boldsymbol{q}'}\delta_{\boldsymbol{p}\boldsymbol{p}'} + \boldsymbol{p}'\cdot\boldsymbol{k}\sum_{\theta}c_{p'}^{\theta\mu}\delta_{\alpha\mu}\delta_{\beta\theta}\delta_{\boldsymbol{q}\boldsymbol{q}'}\delta_{\boldsymbol{p}\boldsymbol{p}'} + \sum_{\epsilon\sigma}\rho c_3^{\epsilon\sigma\mu}(\boldsymbol{q}',\boldsymbol{p}')k^2x_\mu\delta_{\alpha\epsilon}\delta_{\beta\sigma}\delta_{\boldsymbol{q}\boldsymbol{q}'}\delta_{\boldsymbol{p}\boldsymbol{p}'}\right]$$

$$= -\frac{i}{2}\sum_{\alpha\beta\boldsymbol{q}} |\rho_{\boldsymbol{q}}^{\alpha}\rho_{\boldsymbol{k}-\boldsymbol{q}}^{\beta})\frac{\rho}{\sqrt{N}}\frac{k_BT}{m_\mu}\left[\boldsymbol{q}\cdot\boldsymbol{k}c_q^{\alpha\mu}\delta_{\beta\mu} + (\boldsymbol{k}-\boldsymbol{q})\cdot\boldsymbol{k}c_{|\boldsymbol{k}-\boldsymbol{q}|}^{\beta\mu}\delta_{\alpha\mu} + \rho k^2x_\mu c_3^{\alpha\beta\mu}(\boldsymbol{q},\boldsymbol{k}-\boldsymbol{q})\right], \tag{27}$$

and, consequently, the memory function yields

$$(f_{\boldsymbol{k}}^{\mu}|f_{\boldsymbol{k}}^{\nu}(t)) = \frac{\rho^2}{4N}\sum_{\alpha\beta\boldsymbol{q}}\sum_{\alpha'\beta'\boldsymbol{q}'}\frac{k_BT}{m_\mu}\mathcal{V}_{\alpha\beta\mu}(\boldsymbol{q},\boldsymbol{k}-\boldsymbol{q},\boldsymbol{k})(\rho_{\boldsymbol{q}}^{\alpha}\rho_{\boldsymbol{k}-\boldsymbol{q}}^{\beta}|\rho_{\boldsymbol{q}'}^{\alpha'}(t)\rho_{\boldsymbol{k}-\boldsymbol{q}'}^{\beta'}(t))\mathcal{V}_{\alpha'\beta'\nu}(\boldsymbol{q}',\boldsymbol{k}-\boldsymbol{q}',\boldsymbol{k})\frac{k_BT}{m_\nu}, \tag{28}$$

with the vertex, which represents the coupling strength between different wavevectors, given by

$$\mathcal{V}_{\alpha\beta\mu}(\boldsymbol{q},\boldsymbol{k}-\boldsymbol{q},\boldsymbol{k}) = \boldsymbol{q}\cdot\boldsymbol{k}c_q^{\alpha\mu}\delta_{\beta\mu} + (\boldsymbol{k}-\boldsymbol{q})\cdot\boldsymbol{k}c_{|\boldsymbol{k}-\boldsymbol{q}|}^{\beta\mu}\delta_{\alpha\mu} + \rho k^2x_\mu c_3^{\alpha\beta\mu}(\boldsymbol{q},\boldsymbol{k}-\boldsymbol{q}). \tag{29}$$

Note that, apart from the unknown dynamic density correlation functions $(\rho_{\boldsymbol{q}}^{\alpha}\rho_{\boldsymbol{k}-\boldsymbol{q}}^{\beta}|\rho_{\boldsymbol{q}'}^{\alpha'}(t)\rho_{\boldsymbol{k}-\boldsymbol{q}'}^{\beta'}(t))$, all other terms in the memory function [Eq. (28)] are static, and in principle known, equilibrium properties of the system.

In standard MCT, the multi-point density correlation functions $(\rho_{\boldsymbol{q}}^{\alpha}\rho_{\boldsymbol{k}-\boldsymbol{q}}^{\beta}|\rho_{\boldsymbol{q}'}^{\alpha'}(t)\rho_{\boldsymbol{k}-\boldsymbol{q}'}^{\beta'}(t))$ are simplified in two steps. The first one is the so-called diagonal approximation, i.e.

$$(\rho_{\boldsymbol{q}}^{\alpha}\rho_{\boldsymbol{k}-\boldsymbol{q}}^{\beta}|\rho_{\boldsymbol{q}'}^{\alpha'}(t)\rho_{\boldsymbol{k}-\boldsymbol{q}'}^{\beta'}(t)) \approx (\rho_{\boldsymbol{q}}^{\alpha}\rho_{\boldsymbol{k}-\boldsymbol{q}}^{\beta}|\rho_{\boldsymbol{q}'}^{\alpha'}(t)\rho_{\boldsymbol{k}-\boldsymbol{q}'}^{\beta'}(t))(\delta_{\boldsymbol{q},\boldsymbol{q}'} + \delta_{\boldsymbol{q},\boldsymbol{k}-\boldsymbol{q}'})$$

$$= (\rho_{\boldsymbol{q}}^{\alpha}\rho_{\boldsymbol{k}-\boldsymbol{q}}^{\beta}|\rho_{\boldsymbol{q}}^{\alpha'}(t)\rho_{\boldsymbol{k}-\boldsymbol{q}}^{\beta'}(t))\delta_{\boldsymbol{q},\boldsymbol{q}'} + (\rho_{\boldsymbol{q}}^{\alpha}\rho_{\boldsymbol{k}-\boldsymbol{q}}^{\beta}|\rho_{\boldsymbol{k}-\boldsymbol{q}}^{\alpha'}(t)\rho_{\boldsymbol{q}}^{\beta'}(t))\delta_{\boldsymbol{q},\boldsymbol{k}-\boldsymbol{q}'}, \tag{30}$$

which, using $\mathcal{V}_{\beta'\alpha'\nu}(\boldsymbol{k}-\boldsymbol{q},\boldsymbol{q},\boldsymbol{k}) = \mathcal{V}_{\alpha'\beta'\nu}(\boldsymbol{q},\boldsymbol{k}-\boldsymbol{q},\boldsymbol{k})$, implies that

$$(f_{\boldsymbol{k}}^{\mu}|f_{\boldsymbol{k}}^{\nu}(t)) = \frac{\rho^2}{4N}\sum_{\alpha\beta\boldsymbol{q}}\sum_{\alpha'\beta'}\frac{k_BT}{m_\mu}\mathcal{V}_{\alpha\beta\mu}(\boldsymbol{q},\boldsymbol{k}-\boldsymbol{q},\boldsymbol{k})(\rho_{\boldsymbol{q}}^{\alpha}\rho_{\boldsymbol{k}-\boldsymbol{q}}^{\beta}|\rho_{\boldsymbol{q}}^{\alpha'}(t)\rho_{\boldsymbol{k}-\boldsymbol{q}}^{\beta'}(t))\mathcal{V}_{\alpha'\beta'\nu}(\boldsymbol{q},\boldsymbol{k}-\boldsymbol{q},\boldsymbol{k})\frac{k_BT}{m_\nu}$$

$$+\frac{\rho^2}{4N}\sum_{\alpha\beta\boldsymbol{q}}\sum_{\alpha'\beta'}\frac{k_BT}{m_\mu}\mathcal{V}_{\alpha\beta\mu}(\boldsymbol{q},\boldsymbol{k}-\boldsymbol{q},\boldsymbol{k})(\rho_{\boldsymbol{q}}^{\alpha}\rho_{\boldsymbol{k}-\boldsymbol{q}}^{\beta}|\rho_{\boldsymbol{k}-\boldsymbol{q}}^{\alpha'}(t)\rho_{\boldsymbol{q}}^{\beta'}(t))\mathcal{V}_{\alpha'\beta'\nu}(\boldsymbol{k}-\boldsymbol{q},\boldsymbol{q},\boldsymbol{k})\frac{k_BT}{m_\nu} \tag{31}$$

$$= \frac{\rho^2}{2N}\sum_{\alpha\beta\boldsymbol{q}}\sum_{\alpha'\beta'}\frac{k_BT}{m_\mu}\mathcal{V}_{\alpha\beta\mu}(\boldsymbol{q},\boldsymbol{k}-\boldsymbol{q},\boldsymbol{k})(\rho_{\boldsymbol{q}}^{\alpha}\rho_{\boldsymbol{k}-\boldsymbol{q}}^{\beta}|\rho_{\boldsymbol{q}}^{\alpha'}(t)\rho_{\boldsymbol{k}-\boldsymbol{q}}^{\beta'}(t))\mathcal{V}_{\alpha'\beta'\nu}(\boldsymbol{q},\boldsymbol{k}-\boldsymbol{q},\boldsymbol{k})\frac{k_BT}{m_\nu}. \tag{32}$$

The second step is Gaussian factorization of the multi-point density correlations,

$$(\rho_{\boldsymbol{q}}^{\alpha}\rho_{\boldsymbol{k}-\boldsymbol{q}}^{\beta}|\rho_{\boldsymbol{q}}^{\alpha'}(t)\rho_{\boldsymbol{k}-\boldsymbol{q}}^{\beta'}(t)) \approx (\rho_{\boldsymbol{q}}^{\alpha}|\rho_{\boldsymbol{q}}^{\alpha'}(t))(\rho_{\boldsymbol{k}-\boldsymbol{q}}^{\beta}|\rho_{\boldsymbol{k}-\boldsymbol{q}}^{\beta'}(t)) = F_{\alpha\alpha'}(\boldsymbol{q},t)F_{\beta\beta'}(\boldsymbol{k}-\boldsymbol{q},t), \tag{33}$$

so that the memory function in standard MCT becomes a function of the 2-point density correlation function,

$$(f_{\boldsymbol{k}}^{\mu}|f_{\boldsymbol{k}}^{\nu}(t)) = \frac{\rho^2}{2N}\sum_{\boldsymbol{q}}\sum_{\alpha\beta\alpha'\beta'}\frac{k_BT}{m_\mu}\mathcal{V}_{\alpha\beta\mu}(\boldsymbol{q},\boldsymbol{k}-\boldsymbol{q},\boldsymbol{k})F_{\alpha\alpha'}(\boldsymbol{q},t)F_{\beta\beta'}(\boldsymbol{k}-\boldsymbol{q},t)\mathcal{V}_{\alpha'\beta'\nu}(\boldsymbol{q},\boldsymbol{k}-\boldsymbol{q},\boldsymbol{k})\frac{k_BT}{m_\nu}$$

$$= \frac{\rho}{2}\int\frac{d\boldsymbol{q}}{(2\pi)^3}\sum_{\alpha\beta\alpha'\beta'}\frac{k_BT}{m_\mu}\mathcal{V}_{\alpha\beta\mu}(\boldsymbol{q},\boldsymbol{k}-\boldsymbol{q},\boldsymbol{k})F_{\alpha\alpha'}(\boldsymbol{q},t)F_{\beta\beta'}(\boldsymbol{k}-\boldsymbol{q},t)\mathcal{V}_{\alpha'\beta'\nu}(\boldsymbol{q},\boldsymbol{k}-\boldsymbol{q},\boldsymbol{k})\frac{k_BT}{m_\nu}, \tag{34}$$

where in the last step we have assumed the thermodynamic limit to write $\sum_{\boldsymbol{q}} = \frac{V}{(2\pi)^3}\int d\boldsymbol{q}$. Overall, the above set of approximations renders Eq. (16) a closed equation for $F_{\alpha\beta}(\boldsymbol{k},t)$ which can be solved self-consistently.

3. Multi-component GMCT

The main idea of GMCT is to avoid the most severe approximation of MCT, namely the factorization approximation of Eq. (33), and instead use an explicit expression for the diagonal 4-point density correlation functions $(\rho^{\alpha}_{\boldsymbol{q}}\rho^{\beta}_{\boldsymbol{k-q}}|\rho^{\alpha'}_{\boldsymbol{q}}(t)\rho^{\beta'}_{\boldsymbol{k-q}}(t))$. More generally, we seek to retrieve the exact equations of motion for arbitrary diagonal $2n$-point density correlation functions $(\rho^{\alpha_1}_{\boldsymbol{k_1}}\rho^{\alpha_2}_{\boldsymbol{k_2}}\cdots\rho^{\alpha_n}_{\boldsymbol{k_n}}|\rho^{\beta_1}_{\boldsymbol{k_1}}(t)\rho^{\beta_2}_{\boldsymbol{k_2}}(t)\cdots\rho^{\beta_n}_{\boldsymbol{k_n}}(t))$, and develop a hierarchy of equations relating them to each other. Following previous work on single component systems [5], we generalize the vector $\boldsymbol{A}$ to include the n-point density modes and associated current modes at a given series of wavevectors $\{\boldsymbol{k}_1,\boldsymbol{k}_2,\cdots,\boldsymbol{k}_n\}$ (we assume $\boldsymbol{k}_i \neq \boldsymbol{k}_j$ for $i\neq j$),

$$\boldsymbol{A} = \left[\left\{\rho^{\alpha_1}_{\boldsymbol{k_1}}\rho^{\alpha_2}_{\boldsymbol{k_2}}\cdots\rho^{\alpha_n}_{\boldsymbol{k_n}}\right\}, -i\left\{\frac{d}{dt}(\rho^{\alpha_1}_{\boldsymbol{k_1}}\rho^{\alpha_2}_{\boldsymbol{k_2}}\cdots\rho^{\alpha_n}_{\boldsymbol{k_n}})\right\}\right] \equiv [\boldsymbol{A}_1,\boldsymbol{A}_2], \tag{35}$$

where we point out that there are M^n elements in both $\boldsymbol{A}_1$ and $\boldsymbol{A}_2$. Employing the same procedure as in the derivation of MCT and realizing that $\boldsymbol{A}_2 = -i\dot{\boldsymbol{A}}_1$ and $(\boldsymbol{A}_1|\boldsymbol{A}_2) = 0$, we can calculate generalized versions of $\boldsymbol{G}$, $(\boldsymbol{A}|\dot{\boldsymbol{A}})$, and $\boldsymbol{f}$ in Eq. (9). In particular, we have (adding the superscript (n) to distinguish between different levels with $n = 1$ referring to MCT),

$$\boldsymbol{G}^{(n)} = \begin{bmatrix} \boldsymbol{S}^{(n)}(\{k_i\}) & \boldsymbol{0} \\ \boldsymbol{0} & \boldsymbol{J}^{(n)}(\{k_i\}) \end{bmatrix}, \tag{36}$$

and

$$(\boldsymbol{A}|\dot{\boldsymbol{A}}) = \begin{bmatrix} \boldsymbol{0} & i\boldsymbol{J}^{(n)}(\{k_i\}) \\ i\boldsymbol{J}^{(n)}(\{k_i\}) & \boldsymbol{0} \end{bmatrix}. \tag{37}$$

Here, $\boldsymbol{S}^{(n)}$ and $\boldsymbol{J}^{(n)}$ are $M^n \times M^n$ matrices with elements

$$\begin{aligned} S^{(n)}_{\{\alpha_i\};\{\beta_i\}}(\{k_i\}) &= (\rho^{\alpha_1}_{\boldsymbol{k_1}}\rho^{\alpha_2}_{\boldsymbol{k_2}}\cdots\rho^{\alpha_n}_{\boldsymbol{k_n}}|\rho^{\beta_1}_{\boldsymbol{k_1}}\rho^{\beta_2}_{\boldsymbol{k_2}}\cdots\rho^{\beta_n}_{\boldsymbol{k_n}}) \\ &\approx (\rho^{\alpha_1}_{\boldsymbol{k_1}}|\rho^{\beta_1}_{\boldsymbol{k_1}})(\rho^{\alpha_2}_{\boldsymbol{k_2}}|\rho^{\beta_2}_{\boldsymbol{k_2}})\cdots(\rho^{\alpha_n}_{\boldsymbol{k_n}}|\rho^{\beta_n}_{\boldsymbol{k_n}}) \\ &= S^{\alpha_1\beta_1}_{k_1}S^{\alpha_2\beta_2}_{k_2}\cdots S^{\alpha_n\beta_n}_{k_n} = \prod_{i=1}^{n} S^{\alpha_i\beta_i}(k_i), \end{aligned} \tag{38}$$

and

$$\begin{aligned} J^{(n)}_{\{\alpha_i\};\{\beta_i\}}(\{k_i\}) &= \left(\frac{d}{dt}(\rho^{\alpha_1}_{\boldsymbol{k_1}}\rho^{\alpha_2}_{\boldsymbol{k_2}}\cdots\rho^{\alpha_n}_{\boldsymbol{k_n}})\middle|\frac{d}{dt}(\rho^{\beta_1}_{\boldsymbol{k_1}}\rho^{\beta_2}_{\boldsymbol{k_2}}\cdots\rho^{\beta_n}_{\boldsymbol{k_n}})\right) \\ &= (\dot{\rho}^{\alpha_1}_{\boldsymbol{k_1}}\rho^{\alpha_2}_{\boldsymbol{k_2}}\cdots\rho^{\alpha_n}_{\boldsymbol{k_n}}|\dot{\rho}^{\beta_1}_{\boldsymbol{k_1}}\rho^{\beta_2}_{\boldsymbol{k_2}}\cdots\rho^{\beta_n}_{\boldsymbol{k_n}}) + \cdots + (\rho^{\alpha_1}_{\boldsymbol{k_1}}\rho^{\alpha_2}_{\boldsymbol{k_2}}\cdots\dot{\rho}^{\alpha_n}_{\boldsymbol{k_n}}|\rho^{\beta_1}_{\boldsymbol{k_1}}\rho^{\beta_2}_{\boldsymbol{k_2}}\cdots\dot{\rho}^{\beta_n}_{\boldsymbol{k_n}}) \\ &\approx (\dot{\rho}^{\alpha_1}_{\boldsymbol{k_1}}|\dot{\rho}^{\beta_1}_{\boldsymbol{k_1}})(\rho^{\alpha_2}_{\boldsymbol{k_2}}|\rho^{\beta_2}_{\boldsymbol{k_2}})\cdots(\rho^{\alpha_n}_{\boldsymbol{k_n}}|\rho^{\beta_n}_{\boldsymbol{k_n}}) + \cdots + (\rho^{\alpha_1}_{\boldsymbol{k_1}}|\rho^{\beta_1}_{\boldsymbol{k_1}})(\rho^{\alpha_2}_{\boldsymbol{k_2}}|\rho^{\beta_2}_{\boldsymbol{k_2}})\cdots(\dot{\rho}^{\alpha_n}_{\boldsymbol{k_n}}|\dot{\rho}^{\beta_n}_{\boldsymbol{k_n}}) \\ &= \delta_{\alpha_1\beta_1}k_1^2\frac{k_BT}{m_{\alpha_1}}x_{\alpha_1}S^{\alpha_2\beta_2}_{k_2}\cdots S^{\alpha_n\beta_n}_{k_n} + \cdots + S^{\alpha_1\beta_1}_{k_1}S^{\alpha_2\beta_2}_{k_2}\cdots\delta_{\alpha_n\beta_n}k_n^2\frac{k_BT}{m_{\alpha_n}}x_{\alpha_n} \\ &= \sum_{i=1}^{n}\delta_{\alpha_i,\beta_i}\frac{k_BTx_{\alpha_i}k_i^2}{m_{\alpha_i}S^{\alpha_i\beta_i}_{k_i}}\prod_{j=1}^{n}S^{\alpha_j\beta_j}_{k_j}, \end{aligned} \tag{39}$$

respectively. Moreover, the generalized fluctuating force is given by

$$\boldsymbol{f} = \left[\dot{\boldsymbol{A}}_1,\dot{\boldsymbol{A}}_2\right] - \left[\boldsymbol{A}_1,\boldsymbol{A}_2\right]\begin{bmatrix} \boldsymbol{S}^{-1}(q) & \boldsymbol{0} \\ \boldsymbol{0} & \boldsymbol{J}^{-1}(q) \end{bmatrix}\begin{bmatrix} \boldsymbol{0} & i\boldsymbol{J}(q) \\ i\boldsymbol{J}(q) & \boldsymbol{0} \end{bmatrix} \tag{40}$$

$$= \left[\dot{\boldsymbol{A}}_1,\dot{\boldsymbol{A}}_2\right] - \left[i\boldsymbol{A}_2, i\boldsymbol{A}_1\boldsymbol{S}^{-1}(q)\boldsymbol{J}(q)\right] \tag{41}$$

$$= \left[\boldsymbol{0},\dot{\boldsymbol{A}}_2 - i\boldsymbol{A}_1\boldsymbol{S}^{-1}(q)\boldsymbol{J}(q)\right], \tag{42}$$

so that its elements are,

$$f^{(n)}_{\{\alpha_i\}}(\{k_i\}) = -i\frac{d^2(\rho^{\alpha_1}_{\boldsymbol{k_1}}\rho^{\alpha_2}_{\boldsymbol{k_2}}\cdots\rho^{\alpha_n}_{\boldsymbol{k_n}})}{dt^2} - i\sum_{\{\beta_i\}\{\gamma_i\}}\rho^{\beta_1}_{\boldsymbol{k_1}}\rho^{\beta_2}_{\boldsymbol{k_2}}\cdots\rho^{\beta_n}_{\boldsymbol{k_n}}\left(S^{(n)}(\{k_i\})\right)^{-1}_{\{\beta_i\};\{\gamma_i\}}J^{(n)}_{\{\gamma_i\}\{\alpha_i\}}(\{k_i\}). \tag{43}$$

Using these terms and the Mori-Zwanzig formalism we can determine the dynamical equation for arbitrary $2n$-point density correlation functions, which yields

$$\ddot{F}^{(n)}_{\{\alpha_i\};\{\beta_i\}}(\{k_i\},t) + \sum_{\{\gamma_i\}\{\theta_i\}} F^{(n)}_{\{\alpha_i\};\{\gamma_i\}}(\{k_i\},t)\left(S^{(n)}\right)^{-1}_{\{\gamma_i\};\{\theta_i\}}(\{k_i\})J^{(n)}_{\{\theta_i\};\{\beta_i\}}(\{k_i\})$$
$$+ \int_0^t d\tau \sum_{\{\gamma_i\}\{\theta_i\}} \dot{F}^{(n)}_{\{\alpha_i\};\{\gamma_i\}}(\{k_i\},t-\tau)\left(J^{(n)}\right)^{-1}_{\{\gamma_i\};\{\theta_i\}}(\{k_i\})K^{(n)}_{\{\theta_i\};\{\beta_i\}}(\{k_i\},\tau) = 0. \quad (44)$$

This serves as the starting point in our main text (see Sec. II). The memory kernel is again a correlation between (generalized) fluctuating forces $K^{(n)}_{\{\theta_i\};\{\beta_i\}}(\{k_i\},\tau) = (f^{(n)}_{\{\theta_i\}}(\{k_i\})|f^{(n)}_{\{\beta_i\}}(\{k_i\},\tau))$ and we approximate it, in the same fashion as MCT, by projecting these forces onto $(n+1)$th order density modes and replacing the orthogonal time evolution with a full one, i.e. $(f^{(n)}_{\{\theta_i\}}(\{k_i\})|f^{(n)}_{\{\beta_i\}}(\{k_i\},\tau)) \approx (f^{(n)}_{\{\theta_i\}}(\{k_i\})P^{(n)}|e^{iL\tau}P^{(n)}f^{(n)}_{\{\beta_i\}}(\{k_i\}))$. Inspired by single component GMCT [5] and the simplified shape of P_2 introduced for MCT, we define the nth order projection operator as the summation of orthogonal projection operators:

$$P^{(n)} = \sum_i P^{(n)}_i \quad (45)$$

with

$$P^{(n)}_i = \sum_{\boldsymbol{q}'\alpha'_0,\cdots,\alpha'_n}^{\boldsymbol{q},\alpha_0,\cdots,\alpha_n} |\rho^{\alpha_0}_{\boldsymbol{k_i}-\boldsymbol{q}}\rho^{\alpha_1}_{\boldsymbol{k_1}}\rho^{\alpha_2}_{\boldsymbol{k_2}}\cdots\rho^{\alpha_{i-1}}_{\boldsymbol{k}_{i-1}}\rho^{\alpha_i}_{\boldsymbol{q}}\rho^{\alpha_{i+1}}_{\boldsymbol{k}_{i+1}}\cdots\rho^{\alpha_n}_{\boldsymbol{k_n}})T^{\boldsymbol{q},\alpha_0,\cdots,\alpha_n}_{\boldsymbol{q}'\alpha'_0,\cdots,\alpha'_n}(i)(\rho^{\alpha'_0}_{\boldsymbol{k_i}-\boldsymbol{q}'}\rho^{\alpha'_1}_{\boldsymbol{k_1}}\rho^{\alpha'_2}_{\boldsymbol{k_2}}\cdots\rho^{\alpha'_{i-1}}_{\boldsymbol{k}_{i-1}}\rho^{\alpha'_i}_{\boldsymbol{q}'}\rho^{\alpha'_{i+1}}_{\boldsymbol{k}_{i+1}}\cdots\rho^{\alpha'_n}_{\boldsymbol{k_n}}|. \quad (46)$$

Note that in this notation $P^{(1)}_1 = P_2$. For $i \neq j$ and using Eq. (23), it can be checked that $P^{(n)}_i P^{(n)}_j \sim S_3(\boldsymbol{k}_i - \boldsymbol{q}, \boldsymbol{q}', \boldsymbol{k}_i)S_3(\boldsymbol{q}, \boldsymbol{k}_j, \boldsymbol{k}_j - \boldsymbol{q}') \sim \frac{1}{N} \to 0$, thus demonstrating that $P^{(n)}_i$ is orthogonal to $P^{(n)}_j$. The normalization $T^{\boldsymbol{q},\alpha_0,\cdots,\alpha_n}_{\boldsymbol{q}'\alpha'_0,\cdots,\alpha'_n}(i)$ is determined via the condition $P^{(n)}_i P^{(n)}_i = P^{(n)}_i$. This gives

$$\sum_{q',\alpha'_0,\alpha'_1,\cdots,\alpha'_n} T^{\boldsymbol{q},\alpha_0,\cdots,\alpha_n}_{\boldsymbol{q}'\alpha'_0,\cdots,\alpha'_n}(i)(\rho^{\alpha'_0}_{\boldsymbol{k_i}-\boldsymbol{q}'}\rho^{\alpha'_1}_{\boldsymbol{k_1}}\cdots\rho^{\alpha'_i}_{\boldsymbol{q}'}\cdots\rho^{\alpha'_n}_{\boldsymbol{k_n}}|\rho^{\beta_0}_{\boldsymbol{k_i}-\boldsymbol{p}}\rho^{\beta_1}_{\boldsymbol{k_1}}\cdots\rho^{\beta_i}_{\boldsymbol{p}}\cdots\rho^{\beta_n}_{\boldsymbol{k_n}}) = \delta_{\boldsymbol{qp}}\delta_{\alpha_0\beta_0}\delta_{\alpha_1\beta_1}\cdots\delta_{\alpha_n\beta_n} \quad (47)$$

and hence

$$\sum_{\boldsymbol{q}',\alpha'_0,\alpha'_1,\cdots,\alpha'_n} T^{\boldsymbol{q},\alpha_0,\cdots,\alpha_n}_{\boldsymbol{q}'\alpha'_0,\cdots,\alpha'_n}(i)$$
$$\times\Big[S^{\alpha'_0\beta_0}_{|\boldsymbol{k}_i-\boldsymbol{q}'|}S^{\alpha'_1\beta_1}_{k_1}\cdots S^{\alpha'_i\beta_i}_{q'}\cdots S^{\alpha'_n\beta_n}_{k_n}\delta_{\boldsymbol{q}'\boldsymbol{p}} + S^{\alpha'_0\beta_i}_{|\boldsymbol{k}_i-\boldsymbol{q}'|}S^{\alpha'_1\beta_1}_{k_1}\cdots S^{\alpha'_i\beta_0}_{q'}\cdots S^{\alpha'_n\beta_n}_{k_n}\delta_{\boldsymbol{p},\boldsymbol{k_i}-\boldsymbol{q}'}\Big] = \delta_{\boldsymbol{qq}'}\delta_{\alpha_0\beta_0}\delta_{\alpha_1\beta_1}\cdots\delta_{\alpha_n\beta_n}, \quad (48)$$

which, using the symmetry property $(\rho^{\alpha'_0}_{\boldsymbol{k_i}-\boldsymbol{q}'}\rho^{\alpha'_1}_{\boldsymbol{k_1}}\cdots\rho^{\alpha'_i}_{\boldsymbol{q}'}\cdots\cdots\rho^{\alpha'_n}_{\boldsymbol{k_n}}| = (\rho^{\alpha'_i}_{\boldsymbol{q}'}\rho^{\alpha'_1}_{\boldsymbol{k_1}}\cdots\rho^{\alpha'_0}_{\boldsymbol{k_i}-\boldsymbol{q}'}\cdots\rho^{\alpha'_n}_{\boldsymbol{k_n}}|$, can be simplified to

$$2\sum_{\alpha'_0,\alpha'_1,\cdots,\alpha'_n} T^{\boldsymbol{q},\alpha_0,\cdots,\alpha_n}_{\boldsymbol{p},\alpha'_0,\cdots,\alpha'_n}(i)S^{\alpha'_0\beta_0}_{|\boldsymbol{k}_i-\boldsymbol{p}|}S^{\alpha'_1\beta_1}_{k_1}\cdots S^{\alpha'_i\beta_i}_{p}\cdots S^{\alpha'_n\beta_n}_{k_n} = \delta_{\boldsymbol{qp}}\delta_{\alpha_0\beta_0}\delta_{\alpha_1\beta_1}\cdots\delta_{\alpha_n\beta_n}. \quad (49)$$

This completes the characterization of the generalized projection operators $P^{(n)}_j$.

Now let us calculate the projected fluctuating force $P^{(n)}_j|f^{(n)}_{\{\mu_i\}}(\{k_i\}))$. In order to calculate the projection of the first term of $f^{(n)}_{\{\mu_i\}}(\{k_i\})$, we require an expression for

$$-i(\rho^{\alpha'_0}_{\boldsymbol{k_j}-\boldsymbol{q}'}\rho^{\alpha'_1}_{\boldsymbol{k_1}}\cdots\rho^{\alpha'_j}_{\boldsymbol{q}'}\cdots\rho^{\alpha'_n}_{\boldsymbol{k_n}}|\frac{d^2}{dt^2}(\rho^{\mu_1}_{\boldsymbol{k_1}}\rho^{\mu_2}_{\boldsymbol{k_2}}\cdots\rho^{\mu_n}_{\boldsymbol{k_n}}))$$
$$= i\Big(\frac{d}{dt}(\rho^{\alpha'_0}_{\boldsymbol{k_j}-\boldsymbol{q}'}\rho^{\alpha'_1}_{\boldsymbol{k_1}}\cdots\rho^{\alpha'_j}_{\boldsymbol{q}'}\cdots\rho^{\alpha'_n}_{\boldsymbol{k_n}})|\frac{d}{dt}(\rho^{\mu_1}_{\boldsymbol{k_1}}\rho^{\mu_2}_{\boldsymbol{k_2}}\cdots\rho^{\mu_n}_{\boldsymbol{k_n}})\Big) \quad (50)$$

Realizing that $(\dot{\rho}^{\alpha}_{\boldsymbol{k}}|\rho^{\beta}_{\boldsymbol{k}}) = 0$, we find only three non-zero contributions to this expression:

1. $i(\dot{\rho}^{\alpha'_0}_{\boldsymbol{k_j}-\boldsymbol{q}'}\rho^{\alpha'_1}_{\boldsymbol{k_1}}\cdots\rho^{\alpha'_j}_{\boldsymbol{q}'}\cdots\rho^{\alpha'_n}_{\boldsymbol{k_n}}|\rho^{\mu_1}_{\boldsymbol{k_1}}\rho^{\mu_2}_{\boldsymbol{k_2}}\cdots\dot{\rho}^{\mu_j}_{\boldsymbol{k_j}}\cdots\rho^{\mu_n}_{\boldsymbol{k_n}}) \approx i\frac{1}{\sqrt{N}}\frac{k_BT}{m_{\mu_j}}(\boldsymbol{k}_j - \boldsymbol{q}')\cdot\boldsymbol{k}_j\delta_{\alpha'_0\mu_j}S^{\alpha'_j\mu_j}_{\boldsymbol{q}'}\prod_{i\neq j}S^{\alpha'_i\mu_i}_{k_i}$
2. $i(\rho^{\alpha'_0}_{\boldsymbol{k_j}-\boldsymbol{q}'}\rho^{\alpha'_1}_{\boldsymbol{k_1}}\cdots\dot{\rho}^{\alpha'_j}_{\boldsymbol{q}'}\cdots\rho^{\alpha'_n}_{\boldsymbol{k_n}}|\rho^{\mu_1}_{\boldsymbol{k_1}}\rho^{\mu_2}_{\boldsymbol{k_2}}\cdots\dot{\rho}^{\mu_j}_{\boldsymbol{k_j}}\cdots\rho^{\mu_n}_{\boldsymbol{k_n}}) \approx i\frac{1}{\sqrt{N}}\frac{k_BT}{m_{\mu_j}}\boldsymbol{q}'\cdot\boldsymbol{k}_j\delta_{\alpha'_j\mu_j}S^{\alpha'_0\mu_j}_{|\boldsymbol{k}_j-\boldsymbol{q}'|}\prod_{i\neq j}S^{\alpha'_i\mu_i}_{k_i}$

3. for $i \neq j$,
$i(\rho^{\alpha'_0}_{\boldsymbol{k_j}-\boldsymbol{q'}}\rho^{\alpha'_1}_{\boldsymbol{k_1}}\cdots\rho^{\alpha'_i}_{\boldsymbol{k_i}}\cdots\rho^{\alpha'_j}_{\boldsymbol{q'}}\cdots\rho^{\alpha'_n}_{\boldsymbol{k_n}})|\rho^{\mu_1}_{\boldsymbol{k_1}}\rho^{\mu_2}_{\boldsymbol{k_2}}\cdots\dot\rho^{\mu'_i}_{\boldsymbol{k_i}}\cdots\rho^{\mu_j}_{\boldsymbol{k_j}}\cdots\rho^{\mu_n}_{\boldsymbol{k_n}}) \approx iS_3^{\alpha'_0\alpha'_j\mu_j}(\boldsymbol{k}_j-\boldsymbol{q}',\boldsymbol{q}',\boldsymbol{k}_j)\delta_{\alpha'_i\mu_i}k_i^2\frac{k_BT}{m_{\mu_i}}x_{\mu_i}\prod_{l\neq i,l\neq j}S^{\alpha'_l\mu_l}_{k_l}$

Thus, the right-hand side of Eq. (50) becomes

$$i\frac{1}{\sqrt{N}}\frac{k_BT}{m_{\mu_j}}\Big[(\boldsymbol{k}_j-\boldsymbol{q}')\cdot\boldsymbol{k}_j\delta_{\alpha'_0\mu_j}S^{\alpha'_j\mu_j}_{\boldsymbol{q}'}\prod_{i\neq j}S^{\alpha'_i\mu_i}_{k_i}+\boldsymbol{q}'\cdot\boldsymbol{k}_j\delta_{\alpha'_j\mu_j}S^{\alpha'_0\mu_j}_{|\boldsymbol{k}_j-\boldsymbol{q}'|}\prod_{i\neq j}S^{\alpha'_i\mu_i}_{k_i}\Big]$$
$$+i\sum_{i\neq j}S_3^{\alpha'_0\alpha'_j\mu_j}(\boldsymbol{k}_j-\boldsymbol{q}',\boldsymbol{q}',\boldsymbol{k}_j)\delta_{\alpha'_i\mu_i}k_i^2\frac{k_BT}{m_{\mu_i}}x_{\mu_i}\prod_{l\neq i,l\neq j}S^{\alpha'_l\mu_l}_{k_l} \quad (51)$$

In comparison, for the projection of the second term of the projected force $f^{(n)}_{\{\mu_i\}}(\{k_i\})$ we need the term

$$-i(\rho^{\alpha'_0}_{\boldsymbol{k_j}-\boldsymbol{q'}}\rho^{\alpha'_1}_{\boldsymbol{k_1}}\cdots\rho^{\alpha'_j}_{\boldsymbol{q'}}\cdots\rho^{\alpha'_n}_{\boldsymbol{k_n}}|\sum_{\{\theta_i\}\{\gamma_i\}}\rho^{\theta_1}_{\boldsymbol{k_1}}\rho^{\theta_2}_{\boldsymbol{k_2}}\cdots\rho^{\theta_n}_{\boldsymbol{k_n}})\left(S^{(n)}(\{k_i\}))^{-1}\right)_{\{\theta_i\};\{\gamma_i\}}J^{(n)}_{\{\gamma_i\}\{\mu_i\}}(\{k_i\})$$
$$=-i\sum_{\{\theta_i\}\{\gamma_i\}}S_3^{\alpha'_0\alpha'_j\theta_j}(\boldsymbol{k}_j-\boldsymbol{q}',\boldsymbol{q}',\boldsymbol{k}_j)\prod_{i\neq j}S^{\alpha'_i\theta_i}_{k_i}\left(\prod_{i=1}^nS^{\theta_i\gamma_i}_{k_i}\right)^{-1}\sum_{i=1}^n\delta_{\gamma_i,\mu_i}\frac{k_BTx_{\gamma_i}k_i^2}{m_{\gamma_i}S^{\gamma_i\mu_i}_{k_i}}\prod_{j=1}^nS^{\gamma_j\mu_j}_{k_j}, \quad (52)$$

where we have applied Gaussian factorization to write

$$(\rho^{\alpha'_0}_{\boldsymbol{k_j}-\boldsymbol{q'}}\rho^{\alpha'_1}_{\boldsymbol{k_1}}\cdots\rho^{\alpha'_j}_{\boldsymbol{q'}}\cdots\rho^{\alpha'_n}_{\boldsymbol{k_n}}|\rho^{\theta_1}_{\boldsymbol{k_1}}\rho^{\theta_2}_{\boldsymbol{k_2}}\cdots\rho^{\theta_n}_{\boldsymbol{k_n}})\approx S_3^{\alpha'_0\alpha'_j\theta_j}(\boldsymbol{k}_j-\boldsymbol{q}',\boldsymbol{q}',\boldsymbol{k}_j)\prod_{i\neq j}S^{\alpha'_i\theta_i}_{k_i}. \quad (53)$$

Combining these results, we find that

$$(\rho^{\alpha'_0}_{\boldsymbol{k_j}-\boldsymbol{q'}}\rho^{\alpha'_1}_{\boldsymbol{k_1}}\cdots\rho^{\alpha'_j}_{\boldsymbol{q'}}\cdots\rho^{\alpha'_n}_{\boldsymbol{k_n}}|f^{(n)}_{\{\mu_i\}}(\{k_i\})=i\frac{1}{\sqrt{N}}\frac{k_BT}{m_{\mu_j}}\Big[(\boldsymbol{k}_j-\boldsymbol{q}')\cdot\boldsymbol{k}_j\delta_{\alpha'_0\mu_j}S^{\alpha'_j\mu_j}_{\boldsymbol{q}'}\prod_{i\neq j}S^{\alpha'_i\mu_i}_{k_i}+\boldsymbol{q}'\cdot\boldsymbol{k}_j\delta_{\alpha'_j\mu_j}S^{\alpha'_0\mu_j}_{|\boldsymbol{k}_j-\boldsymbol{q}'|}\prod_{i\neq j}S^{\alpha'_i\mu_i}_{k_i}\Big]$$
$$-i\sum_{\{\theta_i\}\{\gamma_i\}}S_3^{\alpha'_0\alpha'_j\theta_j}(\boldsymbol{k}_j-\boldsymbol{q}',\boldsymbol{q}',\boldsymbol{k}_j)\prod_{i\neq j}S^{\alpha'_i\theta_i}_{k_i}\left(\prod_{i=1}^nS^{\theta_i\gamma_i}_{k_i}\right)^{-1}\delta_{\gamma_j,\mu_j}\frac{k_BTx_{\gamma_j}k_j^2}{m_{\gamma_j}}\prod_{l\neq j}^nS^{\gamma_l\mu_l}_{k_l}$$
$$=i\frac{1}{\sqrt{N}}\frac{k_BT}{m_{\mu_j}}\Big[(\boldsymbol{k}_j-\boldsymbol{q}')\cdot\boldsymbol{k}_j\delta_{\alpha'_0\mu_j}S^{\alpha'_j\mu_j}_{\boldsymbol{q}'}\prod_{i\neq j}S^{\alpha'_i\mu_i}_{k_i}+\boldsymbol{q}'\cdot\boldsymbol{k}_j\delta_{\alpha'_j\mu_j}S^{\alpha'_0\mu_j}_{|\boldsymbol{k}_j-\boldsymbol{q}'|}\prod_{i\neq j}S^{\alpha'_i\mu_i}_{k_i}$$
$$-\sum_{\{\theta_i\}\{\gamma_i\}}\sum_{\epsilon\sigma\eta}S^{\alpha'_0\epsilon}_{|\boldsymbol{k}_j-\boldsymbol{q}'|}S^{\alpha'_j\sigma}_{q'}S^{\theta_j\eta}_{k_j}[\delta_{\epsilon\sigma}\delta_{\sigma\eta}\delta_{\epsilon\eta}/x_\epsilon^2+\rho^2c_3^{\epsilon\sigma\eta}(\boldsymbol{k}_j-\boldsymbol{q}',\boldsymbol{q}')]\prod_{i\neq j}S^{\alpha'_i\theta_i}_{k_i}\left(\prod_{i=1}^nS^{\theta_i\gamma_i}_{k_i}\right)^{-1}\delta_{\gamma_j,\mu_j}x_{\gamma_j}k_j^2\prod_{l\neq j}^nS^{\gamma_l\mu_l}_{k_l}\Big]$$
$$=i\frac{1}{\sqrt{N}}\frac{k_BT}{m_{\mu_j}}\Big[(\boldsymbol{k}_j-\boldsymbol{q}')\cdot\boldsymbol{k}_j\delta_{\alpha'_0\mu_j}S^{\alpha'_j\mu_j}_{\boldsymbol{q}'}\prod_{i\neq j}S^{\alpha'_i\mu_i}_{k_i}+\boldsymbol{q}'\cdot\boldsymbol{k}_j\delta_{\alpha'_j\mu_j}S^{\alpha'_0\mu_j}_{|\boldsymbol{k}_j-\boldsymbol{q}'|}\prod_{i\neq j}S^{\alpha'_i\mu_i}_{k_i}$$
$$-\sum_{\{\gamma_i\}}\sum_{\epsilon\sigma\eta}S^{\alpha'_0\epsilon}_{|\boldsymbol{k}_j-\boldsymbol{q}'|}S^{\alpha'_j\sigma}_{q'}[\delta_{\epsilon\sigma}\delta_{\sigma\eta}\delta_{\epsilon\eta}/x_\epsilon^2+\rho^2c_3^{\epsilon\sigma\eta}(\boldsymbol{k}_j-\boldsymbol{q}',\boldsymbol{q}')]\prod_{i\neq j}\delta_{\alpha'_i\gamma_i}\delta_{\eta\gamma_j}\delta_{\gamma_j\mu_j}x_{\gamma_j}k_j^2\prod_{l\neq j}^nS^{\gamma_l\mu_l}_{k_l}\Big]$$
$$=i\frac{1}{\sqrt{N}}\frac{k_BT}{m_{\mu_j}}\Big[(\boldsymbol{k}_j-\boldsymbol{q}')\cdot\boldsymbol{k}_j\delta_{\alpha'_0\mu_j}S^{\alpha'_j\mu_j}_{\boldsymbol{q}'}\prod_{i\neq j}S^{\alpha'_i\mu_i}_{k_i}+\boldsymbol{q}'\cdot\boldsymbol{k}_j\delta_{\alpha'_j\mu_j}S^{\alpha'_0\mu_j}_{|\boldsymbol{k}_j-\boldsymbol{q}'|}\prod_{i\neq j}S^{\alpha'_i\mu_i}_{k_i}$$
$$-\sum_{\epsilon\sigma}S^{\alpha'_0\epsilon}_{|\boldsymbol{k}_j-\boldsymbol{q}'|}S^{\alpha'_j\sigma}_{q'}[\delta_{\epsilon\sigma}\delta_{\sigma\mu_j}\delta_{\epsilon\mu_j}/x_\epsilon^2+\rho^2c_3^{\epsilon\sigma\mu_j}(\boldsymbol{k}_j-\boldsymbol{q}',\boldsymbol{q}')]x_{\mu_j}k_j^2\prod_{i\neq j}^nS^{\alpha'_i\mu_i}_{k_i}\Big]$$
$$=i\frac{1}{\sqrt{N}}\frac{k_BT}{m_{\mu_j}}\Big[(\boldsymbol{k}_j-\boldsymbol{q}')\cdot\boldsymbol{k}_j\delta_{\alpha'_0\mu_j}S^{\alpha'_j\mu_j}_{\boldsymbol{q}'}\prod_{i\neq j}S^{\alpha'_i\mu_i}_{k_i}+\boldsymbol{q}'\cdot\boldsymbol{k}_j\delta_{\alpha'_j\mu_j}S^{\alpha'_0\mu_j}_{|\boldsymbol{k}_j-\boldsymbol{q}'|}\prod_{i\neq j}S^{\alpha'_i\mu_i}_{k_i}$$
$$-S^{\alpha'_0\mu_j}_{|\boldsymbol{k}_j-\boldsymbol{q}'|}S^{\alpha'_j\mu_j}_{q'}k_j^2\prod_{i\neq j}^nS^{\alpha'_i\mu_i}_{k_i}/x_{\mu_j}-\sum_{\epsilon\sigma}S^{\alpha'_0\epsilon}_{|\boldsymbol{k}_j-\boldsymbol{q}'|}S^{\alpha'_j\sigma}_{q'}\rho^2c_3^{\epsilon\sigma\mu_j}(\boldsymbol{k}_j-\boldsymbol{q}',\boldsymbol{q}')x_{\mu_j}k_j^2\prod_{i\neq j}^nS^{\alpha'_i\mu_i}_{k_i}\Big]$$
$$=-i\frac{\rho}{\sqrt{N}}\frac{k_BT}{m_{\mu_j}}\Big[(\boldsymbol{k}_j-\boldsymbol{q}')\cdot\boldsymbol{k}_j\sum_\theta S^{\alpha'_0\theta}_{|\boldsymbol{k}_j-\boldsymbol{q}'|}S^{\alpha'_j\mu_j}_{\boldsymbol{q}'}\prod_{i\neq j}S^{\alpha'_i\mu_i}_{k_i}c^{\theta\mu_j}_{|\boldsymbol{k}_j-\boldsymbol{q}'|}+\boldsymbol{q}'\cdot\boldsymbol{k}_j\sum_\theta S^{\alpha'_j\theta}_{q'}S^{\alpha'_0\mu_j}_{|\boldsymbol{k}_j-\boldsymbol{q}'|}\prod_{i\neq j}S^{\alpha'_i\mu_i}_{k_i}c^{\theta\mu_j}_{q'}$$
$$+\sum_{\epsilon\sigma}S^{\alpha'_0\epsilon}_{|\boldsymbol{k}_j-\boldsymbol{q}'|}S^{\alpha'_j\sigma}_{q'}\rho^2c_3^{\epsilon\sigma\mu_j}(\boldsymbol{k}_j-\boldsymbol{q}',\boldsymbol{q}')x_{\mu_j}k_j^2\prod_{i\neq j}^nS^{\alpha'_i\mu_i}_{k_i}\Big]. \quad (54)$$

Invoking the normalization, Eq. (49), the projected fluctuating force can then be written as

$$
\begin{aligned}
& P_j^{(n)}|f^{(n)}_{\{\mu_i\}}(\{k_i\}) \\
&= \sum_{\boldsymbol{q}'\alpha_0',\cdots,\alpha_n'}^{\boldsymbol{q},\alpha_0,\cdots,\alpha_n} |\rho^{\alpha_0}_{\boldsymbol{k_j-q}}\rho^{\alpha_1}_{\boldsymbol{k_1}}\cdots\rho^{\alpha_j}_{\boldsymbol{q}}\cdots\rho^{\alpha_n}_{\boldsymbol{k_n}})T^{\boldsymbol{q},\alpha_0,\cdots,\alpha_n}_{\boldsymbol{q}'\alpha_0',\cdots,\alpha_n'}(j)(\rho^{\alpha_0'}_{\boldsymbol{k_j-q'}}\rho^{\alpha_1'}_{\boldsymbol{k_1}}\cdots\rho^{\alpha_j'}_{\boldsymbol{q'}}\cdots\rho^{\alpha_n'}_{\boldsymbol{k_n}}|f^{(n)}_{\{\mu_i\}}(\{k_i\}) \\
&= -i\frac{\rho}{\sqrt{N}}\frac{k_BT}{m_{\mu_j}}\sum_{\boldsymbol{q}'\alpha_0',\cdots,\alpha_n'}^{\boldsymbol{q},\alpha_0,\cdots,\alpha_n} |\rho^{\alpha_0}_{\boldsymbol{k_j-q}}\rho^{\alpha_1}_{\boldsymbol{k_1}}\cdots\rho^{\alpha_j}_{\boldsymbol{q}}\cdots\rho^{\alpha_n}_{\boldsymbol{k_n}})T^{\boldsymbol{q},\alpha_0,\cdots,\alpha_n}_{\boldsymbol{q}'\alpha_0',\cdots,\alpha_n'}(j) \\
& \Big[(\boldsymbol{k}_j-\boldsymbol{q}')\cdot\boldsymbol{k}_j\sum_\theta S^{\alpha_0'\theta}_{|\boldsymbol{k}_j-\boldsymbol{q}'|}S^{\alpha_j'\mu_j}_{\boldsymbol{q}'}\prod_{i\neq j}S^{\alpha_i'\mu_i}_{k_i}c^{\theta\mu_j}_{|\boldsymbol{k}_j-\boldsymbol{q}'|}+\boldsymbol{q}'\cdot\boldsymbol{k}_j\sum_\theta S^{\alpha_j'\theta}_{q'}S^{\alpha_0'\mu_j}_{|\boldsymbol{k}_j-\boldsymbol{q}'|}\prod_{i\neq j}S^{\alpha_i'\mu_i}_{k_i}c^{\theta\mu_j}_{q'} \\
& +\sum_{\epsilon\sigma}S^{\alpha_0'\epsilon}_{|\boldsymbol{k}_j-\boldsymbol{q}'|}S^{\alpha_j'\sigma}_{q'}\rho^2c_3^{\epsilon\sigma\mu_j}(\boldsymbol{k}_j-\boldsymbol{q}',\boldsymbol{q}')x_{\mu_j}k_j^2\prod_{i\neq j}^{n}S^{\alpha_i'\mu_i}_{k_i}\Big] \\
&= -i\frac{\rho}{2\sqrt{N}}\frac{k_BT}{m_{\mu_j}}\sum^{\boldsymbol{q},\alpha_0,\cdots,\alpha_n}|\rho^{\alpha_0}_{\boldsymbol{k_j-q}}\rho^{\alpha_1}_{\boldsymbol{k_1}}\cdots\rho^{\alpha_j}_{\boldsymbol{q}}\cdots\rho^{\alpha_n}_{\boldsymbol{k_n}}) \\
& \Big[(\boldsymbol{k}_j-\boldsymbol{q})\cdot\boldsymbol{k}_j\delta_{\alpha_j\mu_j}\prod_{i\neq j}\delta_{\alpha_i\mu_i}c^{\alpha_0\mu_j}_{|\boldsymbol{k}_j-\boldsymbol{q}'|}+\boldsymbol{q}\cdot\boldsymbol{k}_j\delta_{\alpha_0\mu_j}\prod_{i\neq j}\delta_{\alpha_i\mu_i}c^{\alpha_j\mu_j}_q+\rho^2c_3^{\alpha_0\alpha_j\mu_j}(\boldsymbol{k}_j-\boldsymbol{q},\boldsymbol{q})x_{\mu_j}k_j^2\prod_{i\neq j}^{n}\delta_{\alpha_i\mu_i}\Big] \\
&= -i\frac{\rho}{2\sqrt{N}}\frac{k_BT}{m_{\mu_j}}\sum^{\boldsymbol{q},\alpha_0,\alpha_j}|\rho^{\alpha_0}_{\boldsymbol{k_j-q}}\rho^{\mu_1}_{\boldsymbol{k_1}}\cdots\rho^{\alpha_j}_{\boldsymbol{q}}\cdots\rho^{\mu_n}_{\boldsymbol{k_n}}) \\
& \Big[(\boldsymbol{k}_j-\boldsymbol{q})\cdot\boldsymbol{k}_j\delta_{\alpha_j\mu_j}c^{\alpha_0\mu_j}_{|\boldsymbol{k}_j-\boldsymbol{q}|}+\boldsymbol{q}\cdot\boldsymbol{k}_j\delta_{\alpha_0\mu_j}c^{\alpha_j\mu_j}_q+\rho^2c_3^{\alpha_0\alpha_j\mu_j}(\boldsymbol{k}_j-\boldsymbol{q},\boldsymbol{q})x_{\mu_j}k_j^2\Big] \\
&= -i\frac{\rho}{2\sqrt{N}}\frac{k_BT}{m_{\mu_j}}\sum^{\boldsymbol{q},\alpha_0,\alpha_j}|\rho^{\alpha_0}_{\boldsymbol{k_j-q}}\rho^{\mu_1}_{\boldsymbol{k_1}}\cdots\rho^{\alpha_j}_{\boldsymbol{q}}\cdots\rho^{\mu_n}_{\boldsymbol{k_n}})\mathcal{V}_{\alpha_j\alpha_0\mu_j}(\boldsymbol{q},\boldsymbol{k_j}-\boldsymbol{q},\boldsymbol{k}_j),
\end{aligned} \tag{55}
$$

such that the memory function simplifies to

$$
\begin{aligned}
& K^{(n)}_{\{\mu_i\};\{\nu_i\}}(\{k_i\},\tau) = (f^{(n)}_{\{\mu_i\}}(\{k_i\})P^{(n)}|e^{i\mathcal{L}t}P^{(n)}f^{(n)}_{\{\nu_i\}}(\{k_i\},\tau)) \\
&= (f^{(n)}_{\{\mu_i\}}(\{k_i\})\sum_l P_l^{(n)}|e^{i\mathcal{L}t}\sum_j P_j^{(n)}f^{(n)}_{\{\nu_i\}}(\{k_i\},\tau)) \\
&= \frac{\rho^2}{4N}\sum_l\sum_j\sum_{\boldsymbol{q},\alpha_0,\alpha_l}\sum_{\boldsymbol{q}',\alpha_0',\alpha_j'}\frac{k_BT}{m_{\mu_l}}\mathcal{V}_{\alpha_l\alpha_0\mu_l}(\boldsymbol{q},\boldsymbol{k_l}-\boldsymbol{q},\boldsymbol{k}_l) \\
& (\rho^{\alpha_0}_{\boldsymbol{k_l-q}}\rho^{\mu_1}_{\boldsymbol{k_1}}\cdots\rho^{\alpha_l}_{\boldsymbol{q}}\cdots\rho^{\mu_n}_{\boldsymbol{k_n}}|\rho^{\alpha_0'}_{\boldsymbol{k_j-q'}}(\tau)\rho^{\nu_1}_{\boldsymbol{k_1}}(\tau)\cdots\rho^{\alpha_j'}_{\boldsymbol{q'}}(\tau)\cdots\rho^{\nu_n}_{\boldsymbol{k_n}}(\tau)) \\
& \mathcal{V}_{\alpha_j'\alpha_0'\nu_j}(\boldsymbol{q}',\boldsymbol{k_j}-\boldsymbol{q}',\boldsymbol{k}_j)\frac{k_BT}{m_{\nu_j}},
\end{aligned} \tag{56}
$$

with the same static vertices as in standard MCT [Eq. (29)]. For convenience we will only focus on the diagonal terms of the dynamical multi-component density correlation functions, i.e.

$$
\begin{aligned}
& (\rho^{\alpha_0}_{\boldsymbol{k_l-q}}\rho^{\mu_1}_{\boldsymbol{k_1}}\cdots\rho^{\alpha_l}_{\boldsymbol{q}}\cdots\rho^{\mu_n}_{\boldsymbol{k_n}}|\rho^{\alpha_0'}_{\boldsymbol{k_j-q'}}(\tau)\rho^{\nu_1}_{\boldsymbol{k_1}}(\tau)\cdots\rho^{\alpha_j'}_{\boldsymbol{q'}}(\tau)\cdots\rho^{\nu_n}_{\boldsymbol{k_n}}(\tau)) \\
&\approx (\rho^{\alpha_0}_{\boldsymbol{k_l-q}}\rho^{\mu_1}_{\boldsymbol{k_1}}\cdots\rho^{\alpha_l}_{\boldsymbol{q}}\cdots\rho^{\mu_n}_{\boldsymbol{k_n}}|\rho^{\alpha_0'}_{\boldsymbol{k}_l\boldsymbol{-q}}(\tau)\rho^{\nu_1}_{\boldsymbol{k_1}}(\tau)\cdots\rho^{\alpha_l'}_{\boldsymbol{q}}(\tau)\cdots\rho^{\nu_n}_{\boldsymbol{k_n}}(\tau))\delta_{\boldsymbol{qq'}}\delta_{lj} \\
& +(\rho^{\alpha_0}_{\boldsymbol{k_l-q}}\rho^{\mu_1}_{\boldsymbol{k_1}}\cdots\rho^{\alpha_l}_{\boldsymbol{q}}\cdots\rho^{\mu_n}_{\boldsymbol{k_n}}|\rho^{\alpha_0'}_{\boldsymbol{q}}(\tau)\rho^{\nu_1}_{\boldsymbol{k_1}}(\tau)\cdots\rho^{\alpha_l'}_{\boldsymbol{k_l-q}}(\tau)\cdots\rho^{\nu_n}_{\boldsymbol{k_n}}(\tau))\delta_{\boldsymbol{q},\boldsymbol{k}_j\boldsymbol{-q'}}\delta_{lj},
\end{aligned} \tag{57}
$$

which implies that the memory function reduces to

$$
\begin{aligned}
&K^{(n)}_{\{\mu_i\};\{\nu_i\}}(\{k_i\},\tau)\\
&=\frac{\rho^2}{4N}\sum_j\sum_{\boldsymbol{q}}\sum_{\alpha_0,\alpha_j,\alpha'_0,\alpha'_j}\frac{k_BT}{m_{\mu_j}}\mathcal{V}_{\alpha_j\alpha_0\mu_j}(\boldsymbol{q},\boldsymbol{k_j}-\boldsymbol{q},\boldsymbol{k}_j)\\
&(\rho^{\alpha_0}_{\boldsymbol{k_j}-\boldsymbol{q}}\rho^{\mu_1}_{\boldsymbol{k_1}}\cdots\rho^{\alpha_j}_{\boldsymbol{q}}\cdots\rho^{\mu_n}_{\boldsymbol{k_n}}|\rho^{\alpha'_0}_{\boldsymbol{k_j}-\boldsymbol{q}}(\tau)\rho^{\nu_1}_{\boldsymbol{k_1}}(\tau)\cdots\rho^{\alpha'_j}_{\boldsymbol{q}}(\tau)\cdots\rho^{\nu_n}_{\boldsymbol{k_n}}(\tau))\\
&\mathcal{V}_{\alpha'_j\alpha'_0\nu_j}(\boldsymbol{q},\boldsymbol{k_j}-\boldsymbol{q},\boldsymbol{k}_j)\frac{k_BT}{m_{\nu_j}}\\
&+\frac{\rho^2}{4N}\sum_j\sum_{\boldsymbol{q}}\sum_{\alpha_0,\alpha_j,\alpha'_0,\alpha'_j}\frac{k_BT}{m_{\mu_j}}\mathcal{V}_{\alpha_j\alpha_0\mu_j}(\boldsymbol{q},\boldsymbol{k_j}-\boldsymbol{q},\boldsymbol{k}_j)\\
&(\rho^{\alpha_0}_{\boldsymbol{k_j}-\boldsymbol{q}}\rho^{\mu_1}_{\boldsymbol{k_1}}\cdots\rho^{\alpha_j}_{\boldsymbol{q}}\cdots\rho^{\mu_n}_{\boldsymbol{k_n}}|\rho^{\alpha'_0}_{\boldsymbol{q}}(\tau)\rho^{\nu_1}_{\boldsymbol{k_1}}(\tau)\cdots\rho^{\alpha'_j}_{\boldsymbol{k_j}-\boldsymbol{q}}(\tau)\cdots\rho^{\nu_n}_{\boldsymbol{k_n}}(\tau))\\
&\mathcal{V}_{\alpha'_j\alpha'_0\nu_j}(\boldsymbol{k_j}-\boldsymbol{q},\boldsymbol{q},\boldsymbol{k}_j)\frac{k_BT}{m_{\nu_j}} \qquad (58)\\
&=\frac{\rho^2}{2N}\sum_j\sum_{\boldsymbol{q}}\sum_{\alpha_0,\alpha_j,\alpha'_0,\alpha'_j}\frac{k_BT}{m_{\mu_j}}\mathcal{V}_{\alpha_j\alpha_0\mu_j}(\boldsymbol{q},\boldsymbol{k_j}-\boldsymbol{q},\boldsymbol{k}_j)\\
&(\rho^{\alpha_0}_{\boldsymbol{k_j}-\boldsymbol{q}}\rho^{\mu_1}_{\boldsymbol{k_1}}\cdots\rho^{\alpha_j}_{\boldsymbol{q}}\cdots\rho^{\mu_n}_{\boldsymbol{k_n}}|\rho^{\alpha'_0}_{\boldsymbol{k_j}-\boldsymbol{q}}(\tau)\rho^{\nu_1}_{\boldsymbol{k_1}}(\tau)\cdots\rho^{\alpha'_j}_{\boldsymbol{q}}(\tau)\cdots\rho^{\nu_n}_{\boldsymbol{k_n}}(\tau))\\
&\mathcal{V}_{\alpha'_j\alpha'_0\nu_j}(\boldsymbol{q},\boldsymbol{k_j}-\boldsymbol{q},\boldsymbol{k}_j)\frac{k_BT}{m_{\nu_j}}\\
&=\frac{\rho}{2}\int\frac{d\boldsymbol{q}}{(2\pi)^3}\sum_j\sum_{\alpha_0,\alpha_j,\alpha'_0,\alpha'_j}\frac{k_BT}{m_{\mu_j}}\mathcal{V}_{\alpha_j\alpha_0\mu_j}(\boldsymbol{q},\boldsymbol{k_j}-\boldsymbol{q},\boldsymbol{k}_j)\\
&(\rho^{\alpha_0}_{\boldsymbol{k_j}-\boldsymbol{q}}\rho^{\mu_1}_{\boldsymbol{k_1}}\cdots\rho^{\alpha_j}_{\boldsymbol{q}}\cdots\rho^{\mu_n}_{\boldsymbol{k_n}}|\rho^{\alpha'_0}_{\boldsymbol{k_j}-\boldsymbol{q}}(\tau)\rho^{\nu_1}_{\boldsymbol{k_1}}(\tau)\cdots\rho^{\alpha'_j}_{\boldsymbol{q}}(\tau)\cdots\rho^{\nu_n}_{\boldsymbol{k_n}}(\tau))\\
&\mathcal{V}_{\alpha'_j\alpha'_0\nu_j}(\boldsymbol{q},\boldsymbol{k_j}-\boldsymbol{q},\boldsymbol{k}_j)\frac{k_BT}{m_{\nu_j}}\\
&=\frac{\rho}{2}\int\frac{d\boldsymbol{q}}{(2\pi)^3}\sum_j\sum_{\alpha_0,\alpha_j,\alpha'_0,\alpha'_j}\frac{k_BT}{m_{\mu_j}}\mathcal{V}_{\alpha_j\alpha_0\mu_j}(\boldsymbol{q},\boldsymbol{k_j}-\boldsymbol{q},\boldsymbol{k}_j)\\
&F^{(n+1)}_{\alpha_0\mu_1\cdots\alpha_j\cdots\mu_n;\alpha'_0\nu_1\cdots\alpha'_j\cdots\nu_n}(\boldsymbol{k_j}-\boldsymbol{q},\boldsymbol{k_1},\cdots,\boldsymbol{q},\cdots,\boldsymbol{k_n},\tau)\\
&\mathcal{V}_{\alpha'_j\alpha'_0\nu_j}(\boldsymbol{q},\boldsymbol{k_j}-\boldsymbol{q},\boldsymbol{k}_j)\frac{k_BT}{m_{\nu_j}}. \qquad (59)
\end{aligned}
$$

Note that in going from the second to the third equality we have made use of $\mathcal{V}_{\alpha_0\alpha_j\mu_j}(\boldsymbol{k_j}-\boldsymbol{q},\boldsymbol{q},\boldsymbol{k}_j)=\mathcal{V}_{\alpha_j\alpha_0\mu_j}(\boldsymbol{q},\boldsymbol{k_j}-\boldsymbol{q},\boldsymbol{k}_j)$. Finally, we can rewrite the memory kernel in its more compact final form,

$$
\begin{aligned}
&K^{(n)}_{\{\alpha_i\};\{\beta_i\}}(\{k_i\},\tau)\\
&=\frac{\rho}{2}\sum_{\mu'\nu'}\sum_{\mu\nu}\int\frac{d\mathbf{q}}{(2\pi)^3}\sum_{j=1}^{n}\frac{k_BT}{m_{\alpha_j}}\mathcal{V}_{\mu'\nu'\alpha_j}(\boldsymbol{q},\boldsymbol{k_j}-\boldsymbol{q},\boldsymbol{k_j})F^{(n+1)}_{\mu',\nu',\{\alpha_i\}/\alpha_j;\mu,\nu,\{\beta_i\}/\beta_j}(\boldsymbol{q},\boldsymbol{k_j}-\boldsymbol{q},\{k_i\}/k_j,\tau)\\
&\mathcal{V}_{\mu\nu\beta_j}(\boldsymbol{q},\boldsymbol{k_j}-\boldsymbol{q},\boldsymbol{k_j})\frac{k_BT}{m_{\beta_j}}\ , \qquad (60)
\end{aligned}
$$

which is the expression presented in the main text [Eq. (6)]. As a final remark, note that the factor c_3 in the vertex [Eq. (29)] is neglected in the main work (also known as the convolution approximation), since c_3 generally does not make a dominant contribution for fragile systems [4].

In order to solve the GMCT equations, we have to close the hierarchy at a finite level $n_{\max}$. In the spirit of mean-field models, we choose to approximate the last level $F^{(n_{\max})}$ in the memory kernel in terms of the lower level correlation functions $F^{(n_{\max}-1)}$ and $F^{(1)}$,

$$
\begin{aligned}
&F^{(n_{\max})}_{\mu',\nu',\{\alpha_i\}/\alpha_j;\mu,\nu,\{\beta_i\}/\beta_j}(\boldsymbol{q},\boldsymbol{k_j}-\boldsymbol{q},\{k_i\}/k_j,\tau)\\
&\approx\frac{1}{n_{\max}-2}\sum_{l\neq j}F^{(n_{\max}-1)}_{\mu',\nu',\{\alpha_i\}/\{\alpha_j,\alpha_l\};\mu,\nu,\{\beta_i\}/\{\beta_j,\beta_l\}}(\boldsymbol{q},\boldsymbol{k_j}-\boldsymbol{q},\{k_i\}/\{k_j,k_l\},\tau)F^{(1)}_{\alpha_l\beta_l}(k_l,\tau). \qquad (61)
\end{aligned}
$$

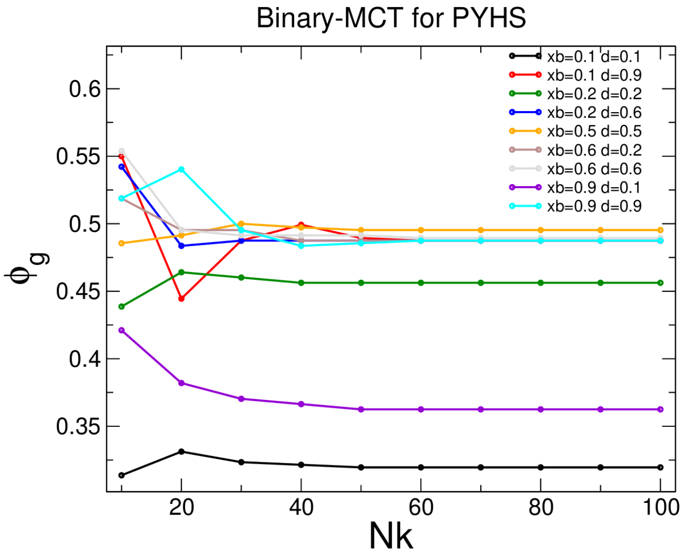

FIG.S 1. Predicted glass-transition packing fraction φ_g as a function of the size of the wavenumber grid, N_k, for binary MCT applied to a binary mixture of hard spheres described by the Percus-Yevick closure. We report different values of the small-particle concentration x_b and the particle size ratio d. Finite size effects appear only around $N_k \sim 60$. We have assumed that a similar N_k convergence holds for binary GMCT applied to LJ and WCA mixtures; in the main manuscript we have used $N_k = 70$.

This leads to the following closure for the memory function at one level lower, i.e. at level $n_{\max} - 1$,

$$K^{(n_{\max}-1)}_{\{\alpha_i\};\{\beta_i\}}(\{k_i\}, t) \approx \frac{1}{n_{\max} - 2} \sum_{j=1}^{n_{\max}-1} K^{(n_{\max}-2)}_{\{\alpha_i\}/\alpha_j;\{\beta_i\}/\beta_j}(\{k_i\}/k_j, t) F^{(1)}_{\alpha_j;\beta_j}(k_j, t). \tag{62}$$

Note that this choice of closure allows us to directly obtain the memory kernel $K^{(n_{\max}-1)}$ from $K^{(n_{\max}-2)}$, which is computationally significantly faster than first estimating $F^{(n_{\max})}$ from Eq. (61) and subsequently calculating $K^{(n_{\max}-1)}$ from Eq. (60).

B. Numerical details

In the main manuscript we report that we used a grid of $N_k = 100$ wavenumbers to solve multi-component GMCT. This value is usually chosen together with a cutoff of $k_{\max} = 40$ to achieve an optimal balance between performance and precision. Ideally, we would like to use a very large N_k, but at the same time we want the computation to remain reasonably fast. This tradeoff becomes more important for complex theories such as multi-component GMCT, where the complexity scales as N_k^n, with n the GMCT order. In Fig. 1 we report the effect of the variation of N_k over the critical packing fraction ϕ_g above which a binary mixture of hard spheres, described using the Percus-Yevick closure, is a glass [6]. Here we have considered different values of the concentration of small particles x_b and the size ratio $d = r_b/r_a$ where r_α is the radius of the hard sphere of species α. Overall, we see that for any binary mixture composition $N_k = 70$ produces the asymptotic value of ϕ_g, and finite size effects are only visible below $N_k = 60$. As a result, a grid of $N_k = 100$ should be amply sufficient to rule out any strong finite size effects. Furthermore, the results of Fig. 1 suggest that even $N_k = 70$ would be acceptable and that it may be worthwhile to use $100 > N_k \geq 70$ to eventually solve GMCT for even higher closure levels n in future studies.

[1] R. Zwanzig, *Nonequilibrium statistical mechanics* (Oxford University Press, 2001).
[2] D. R. Reichman and P. Charbonneau, Journal of Statistical Mechanics: Theory and Experiment **2005**, P05013 (2005).
[3] J. L. Barrat, J. P. Hansen, and G. Pastore, Molecular Physics **63**, 747 (1988).
[4] F. Sciortino and W. Kob, Physical Review Letters **86**, 648 (2001).
[5] L. M. C. Janssen and D. R. Reichman, Physical Review Letters **115**, 205701 (2015).
[6] W. Götze and T. Voigtmann, Physical Review E **67**, 021502 (2003).

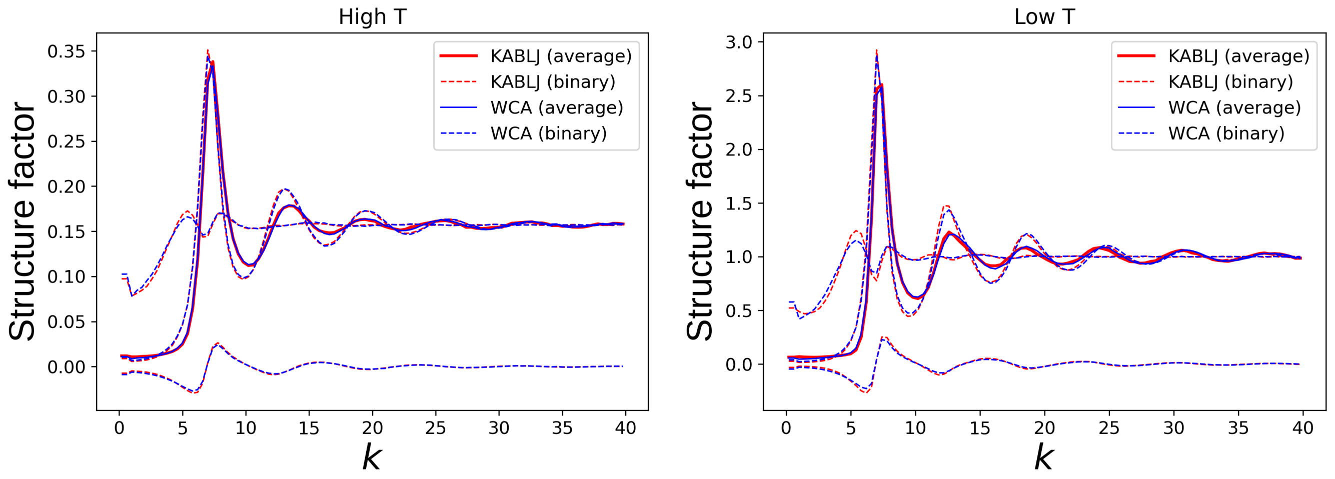

FIG.S 2. Partial structure factors of the binary KABLJ and WCA mixtures are compared to their corresponding averaged monodisperse structure factors. These results are used for the analysis reported in Fig. 2 of the main text. An inspection of both panels demonstrates that averaging not only washes out some of the differences between WCA and KABLJ, but also reduces the effect of the temperature. The latter is exemplified by the weaker growth of the main peak for the averaged monodisperse structure factor at low temperature in comparison to the dominant binary one (i.e. S^{AA}).

**Model**	**Theory**	ρ	$\mathbf{T_0}$	γ	$\mathbf{A_0}$
KABLJ	simulations	1.2	2.7018	0.5478	0.0000
KABLJ	(G)MCT $n_{\max}$ = 2	1.2	1.2235	0.6994	0.5270
KABLJ	GMCT $n_{\max}$ = 3	1.2	1.5285	0.5876	0.7260
KABLJ	GMCT $n_{\max}$ = 4	1.2	2.0671	0.7302	0.8719
WCA	simulations	1.2	5.0175	0.5921	0.0000
WCA	(G)MCT $n_{\max}$ = 2	1.2	1.4513	0.6057	0.6419
WCA	GMCT $n_{\max}$ = 3	1.2	2.0392	0.6652	0.7768
WCA	GMCT $n_{\max}$ = 4	1.2	2.5859	0.6503	0.9677
KABLJ	simulations	1.4	1.8418	1.4885	0.4445
KABLJ	(G)MCT $n_{\max}$ = 2	1.4	0.6263	1.2159	0.8943
KABLJ	GMCT $n_{\max}$ = 3	1.4	0.7779	1.0230	0.8229
KABLJ	GMCT $n_{\max}$ = 4	1.4	0.9000	0.8001	1.1799
WCA	simulations	1.4	2.5831	1.6724	0.4969
WCA	(G)MCT $n_{\max}$ = 2	1.4	0.6555	1.1156	0.8811
WCA	GMCT $n_{\max}$ = 3	1.4	0.8725	1.1153	1.0243
WCA	GMCT $n_{\max}$ = 4	1.4	0.9561	0.7442	1.2418

TABLE S I. The table contains the fitting parameters used to produce Fig. 4 (main manuscript).
